# Approximate, simultaneous comparison of microbial genome architectures via syntenic anchoring of quiver representations

**DOI:** 10.1093/bioinformatics/bty614

**Published:** 2018-09-08

**Authors:** Alex N Salazar, Thomas Abeel

**Affiliations:** 1 Delft Bioinformatics Lab, Delft University of Technology, Delft, The Netherlands; 2 Infectious Disease and Microbiome Program, Broad Institute of MIT and Harvard, Cambridge, MA, USA

## Abstract

**Motivation:**

A long-standing limitation in comparative genomic studies is the dependency on a reference genome, which hinders the spectrum of genetic diversity that can be identified across a population of organisms. This is especially true in the microbial world where genome architectures can significantly vary. There is therefore a need for computational methods that can simultaneously analyze the architectures of multiple genomes without introducing bias from a reference.

**Results:**

In this article, we present Ptolemy: a novel method for studying the diversity of genome architectures—such as structural variation and pan-genomes—across a collection of microbial assemblies without the need of a reference. Ptolemy is a ‘top-down’ approach to compare whole genome assemblies. Genomes are represented as labeled multi-directed graphs—known as quivers—which are then merged into a single, canonical quiver by identifying ‘gene anchors’ via synteny analysis. The canonical quiver represents an approximate, structural alignment of all genomes in a given collection encoding structural variation across (sub-) populations within the collection. We highlight various applications of Ptolemy by analyzing structural variation and the pan-genomes of different datasets composing of *Mycobacterium*, *Saccharomyces, Escherichia* and *Shigella* species. Our results show that Ptolemy is flexible and can handle both conserved and highly dynamic genome architectures. Ptolemy is user-friendly—requires only FASTA-formatted assembly along with a corresponding GFF-formatted file—and resource-friendly—can align 24 genomes in ∼10 mins with four CPUs and <2 GB of RAM.

**Availability and implementation:**

Github: https://github.com/AbeelLab/ptolemy

**Supplementary information:**

Supplementary data are available at *Bioinformatics* online.

## 1 Introduction

Single-molecule sequencing technology has enabled near-complete reconstruction of microbial genomes in both bacterial and eukaryotic organisms (Loman *et al.*, 2015; Rodríguez *et al.*, 2015; Salazar *et al.*, 2017; Yue *et al.*, 2017). Furthermore, ultra-long reads—such as those obtained from Oxford Nanopore Sequencing Technology—can greatly facilitate completion of genome assemblies (Jain *et al.*, 2018). This information enables a more comprehensive understanding of the genomic architecture, variation, and evolution of microbial species (Rodríguez *et al.*, 2015; Salazar *et al.*, 2017; Yue *et al.*, 2017). As single molecule sequencing technologies become more accessible, high-quality microbial assemblies are expected to become more prevalent, decreasing the dependency of a reference genome in comparative studies and instead shifting towards direct assembly-to-assembly analysis.

In general, comparative genomic studies aim to identify differences and similarities in the genetic content of a collection of genomes. Depending on the nature of the research question, this can be achieved via two strategies: ‘bottom-up’ and ‘top-down’. Bottom-up approaches are essentially (multiple) whole genome alignment which use short sub-sequences to anchor and align genomes and which then undergo (multiple) sequence alignment (Angiuoli and Salzberg, 2011; Darling *et al.*, 2010; Kurtz *et al.*, 2004; Paten *et al.*, 2008, 2011). One classic tool is MUMmer (Kurtz *et al.*, 2004), which aligns a query genome to a reference genome using maximal unique matches (MUMs). Clustering of MUMs can then highlight structural differences—such as translocation, inversions, large insertions and deletions—between the query and reference (Kurtz *et al.*, 2004). Sequencing projects dealing with collections of (novel) assemblies often use MUMmer to align the genomes to a common reference and identify variations across the collection of genomes by globally comparing differences between each query and reference (Jain *et al.*, 2018; Salazar *et al.*, 2017; Tyson *et al.*, 2018; Yue *et al.*, 2017). However, these comparisons are biased because these variants only account for differences in sequence that is shared between the query and reference genome. More specifically, nested variation—such as unique sequences in a collection of genomes that are absent in the reference but themselves contain additional variation among each other—are missed.

Multiple-whole genome alignment approaches offer higher resolution of nested variation that can exists across a collection of genomes. Tools like the EPO pipeline (Paten *et al.*, 2008), Cactus (Paten *et al.*, 2011), ProgressiveMauve (Darling *et al.*, 2010) and Mugsy (Angiuoli and Salzberg, 2011), utilize anchor-sequence-finding methods (e.g. MUMs) across a set of genomes to identify collinear regions and thereafter induce multiple sequence alignments across those regions. These approaches are particularly useful in identifying single nucleotide variants and insertion and deletions across several assemblies without bias of a reference. In particular, ProgressiveMauve and Mugsy have been designed in the context of microbial assemblies with ProgressiveMauve tolerating structural variation—such as inversion—common in microbial species (Angiuoli and Salzberg, 2011; Darling *et al.*, 2010); enabling both sequence and structural variation discovery across a collection of genomes. Nevertheless, a major limitation of these approaches is scalability as they have run-times that can take several hours/days depending on genome divergence (Angiuoli and Salzberg, 2011; Darling *et al.*, 2010).

Alternatively, the ‘top-down’ approach in comparing assemblies uses pre-defined biological features as opposed to raw DNA sequence. One widely studied approach is synteny analysis: using gene annotations to identify sets of (coding) sequences that are similar/different across a set of genomes (Ghiurcuta and Moret, 2014). The intuition is that (evolutionary) closely related genomes are not random and instead share a similar genomic structure—such as gene order—due to some common ancestor. The aim is then to identify orthologous sequences across two or more genomes and find segments that maximally extend the collinearity of the gene order, often referred to as synteny. Tools like i-ADHore (Proost *et al.*, 2012), Proteny (Gehrmann and Reinders, 2015), SynFind (Tang *et al.*, 2015) and SynChro (Drillon *et al.*, 2014) aim to identify syntenic regions across a collection of two or more genomes which can then be processed down-stream for further characterization. It is important to note that these methods heavily rely on pre-defined gene annotations and are therefore sensitive to annotation errors. Furthermore, syntenic regions are computationally less expensive to compute since the annotations—equivalent to sequence anchors in methods using the bottom-up approach—are pre-defined. Because the goal of these methods is to compare genomes in terms of gene-order and content, the analysis is generally restricted within one or several syntenic regions (Drillon *et al.*, 2014; Gehrmann and Reinders, 2015; Proost *et al.*, 2012; Tang *et al.*, 2015).

The use of graph-based data structures for comparing multiple genomes has recently been highlighted. More specifically, the paradigm of computational pan-genomics aims to combine multiple assemblies into a single, graph-based data structure to reduce reference bias and enable more robust analysis of variation that exists within a (sub-)population (Marschall *et al.*, 2018). The benefit of this approach has been demonstrated in alignment and variant calling analysis of short-read datasets (Garrison *et al.*, 2017; Rakocevic *et al.*, 2017). In these studies, existing variation were integrated into a common reference genome represented as a graph, which facilitated better placements of short-reads to difficult regions (e.g. highly variable regions), providing a better understanding of the allele composition of those regions within (sub-)populations (Garrison *et al.*, 2017; Rakocevic *et al.*, 2017).

Implementations of graph-based data structures in comparative genomics are not new and have been previously used for a wide-range of genome analysis applications. In terms of microbial genome comparison, the utilization of graph-based representations have been used to compare multiple genome assemblies using a combination of the ‘bottom-up’ (DNA sequence based) and ‘top-down’ (synteny/gene annotation-based) approach. Sibelia, e.g. concatenates multiple genomes sequentially into a single ‘virtual’ genome which is then decomposed into a DNA sequence-based k-mer de Bruijn graph (Minkin *et al.*, 2013). Sets of nodes that are sequentially identically ‘labeled’ (e.g. kmer sequence) are merged thus leading to an alignment de Bruijn (A-Bruijn) graph data structure (Minkin *et al.*, 2013). DRIMM-synteny (Pham and Pevzner, 2010)—a predecessor of Sibelia—uses a similar approach except that it works at the gene-level: nodes are genes, kmers consist of the alphabet of assigned gene labels, and the A-Bruijn graph is constructed by applying the ‘gluing’ operation on identical labeled kmers. Similarly, Pandaconda (Warren *et al.*, 2017) uses pre-assigned family protein labels across multiple genomes, decomposes the genomes into a de Bruijn graph, and applies the gluing operation on identically labeled nodes. Therefore, genetic variation—encoded as alternate paths of genes and gene families—highlight architectural differences across multiple genomes. A major difference is that Pandaconda does not modify the graph to remove cycles enabling discovery of complex structural variations across a set of genomes (Minkin *et al.*, 2013; Pham and Pevzner, 2010; Warren *et al.*, 2017). It is also important to note that both DRIMM-synteny and Pandaconda—which used the ‘top-down’ approach—require pre-assigned labels such that genes that are considered to be identical (e.g. orthologous) have the same label (Pham and Pevzner, 2010; Warren *et al.*, 2017). Ultimately, these graph-based approaches aim to summarize the genetic content of multiple genomes in a single graph data structure to identify genetic variation across multiples assemblies; attempting to place biological context surrounding variation that exists across the genomes.

Here, we present Ptolemy: a method to simultaneously compare the genome architectures of collections of microbial assemblies using both gene synteny and sequence information. Ptolemy is a graph-based and gene annotation approach to aligning multiple genomes (e.g. ‘top-down’), similar to the A-Bruijn methods previously mentioned. However, Ptolemy does not require pre-assigned gene labels and instead computes these labels by identifying maximally syntenic-ortholog-clusters of sequences based on the corresponding gene annotations of an assembly. Furthermore, Ptolemy represents the assemblies via a labeled multi-digraph model (also known as quivers) and uses subsequent morphism mappings to align multiple genomes into a canonical quiver. The resulting representation thus captures structural across a collection of genomes into a single graph data structure which can then be extracted using dynamic maximally labeled path traversal and intuitively visualized with available graph visualization software.

## 2 Materials and methods

The algorithms for our graph and synteny-based approach for simultaneous alignment of multiple genomes is packaged into Ptolemy and takes as input a set of FASTA-formatted assemblies along with their gene annotations in GFF-format. The two novel contributions of Ptolemy are the genome representation and corresponding utilities of labeled multi-digraph, known as quivers (Derksen and Weyman, 2005; Savage, 2006), and the syntenic-anchor finding algorithm. In the following sections, we provide a detailed description of the algorithms used in Ptolemy: first, we describe the quiver representation of a genome and morphism mappings to structurally align multiple genomes without the need of a reference via a ‘top-down’ approach (e.g. orthologous genes). We then describe our implementation of constructing such representation using syntenic-anchors based on syteny-based ortholog clustering. Finally, we describe how structural variation can be extracted from the quiver as a population using dynamic path traversal of labeled edges.

### 2.1 Synteny and the quiver representation of genomes

As previously mentioned, synteny analysis exploits the property that the locations of genes in evolutionary close genome are not random but instead share common structures such as gene order (Drillon *et al.*, 2014; Gehrmann and Reinders, 2015; Ghiurcuta and Moret, 2014; Kuzniar *et al.*, 2008; Poyatos and Hurst, 2007; Proost *et al.*, 2012; Tang *et al.*, 2008, 2015). The term *ortholog* has been used to describe gene sequences between two genomes that derived from a common ancestral gene due to strain/species deviation (Fitch, 2000; Kuzniar *et al.*, 2008). Intuitively, two closely related genomes will retain a large fraction of orthologs along with the order of which they appear throughout the genome, referred to as *synteny* (Duran *et al.*, 2009). Over time, structural variation (such as gene duplications and deletions) and chromosomal rearrangements (including translocations, inversions and horizontal gene transfer) disrupts synteny between genomes (Duran *et al.*, 2009). These disruptions are therefore indicative of structural variation (Duran *et al.*, 2009; Warren *et al.*, 2017).

**Fig. 1.**
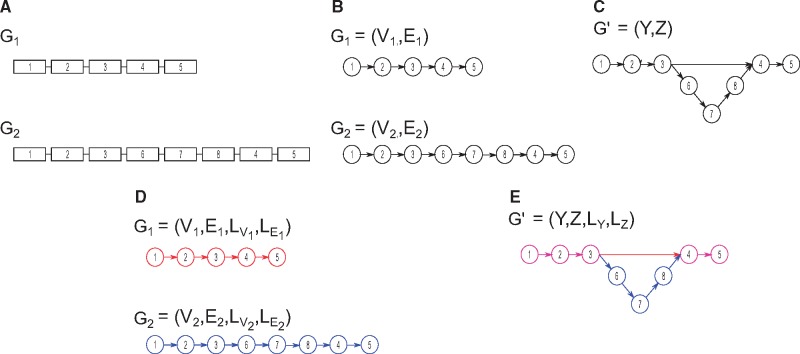
Representing genome architectures as graphs. Panel **(A)** shows two genomes, G_1_ and G_2_, each containing a single chromosome with five to eight genes. Panel **(B)** shows graphical representation of genomes G_1_ and G_2_. Merging similar nodes in the genome graphs shown previously results in a new graph, **(C)**. The addition of labels to nodes and edges results in a labeled multi-directed graph, also known as quivers. Panel **(D)** shows the quiver representation for genomes G_1_ and G_2_—in this case the colors correspond to the labels of G_1_ and G_2_. Merging of the two quivers similarly results in **(E)**, the canonical quiver representation of genomes G_1_ and G_2_

Under the context of a directed graph, the disruption of synteny would induce alternate paths between genomes. Let a genome, G, be represented as a graph, G=(V,E), where the vertex set V contains all genes in G. The edge-set, E, is a set of directed edges describing the order of genes in a genome (e.g. left to right) such that two adjacent genes v and w are connected by a directed edge, e, describing v→w. Note that this high-level graph representation of a genome will contain disconnected components each corresponding to a chromosome. Now, imagine a working example of two closely related genomes, $G_{1}\text{ and}G_{2}$ (see Fig. 1). Constructing the high-level representation of both genomes will yield nearly identical graphs with the exception of topological differences associated with structural variation (see Fig. 1B). By merging identical nodes and edges—which in this context corresponds to orthologous genes in genomes $G_{1}\text{ and }G_{2}$—we create a single, canonical genome graph, $G^{\prime }$, for both genomes, naturally inducing alternate paths that reflect structural variation (see Fig. 1C).

The addition of labels to nodes and edges to the directed graph representations of genomes $G_{1}\text{ and }G_{2}$ results in a labeled multi-directed graph, also known as a quiver (see Fig. 1D) (Derksen and Weyman, 2005; Savage, 2006). Formally, a *quiver* of a genome G, is a graph, $G=(V,E,L_{V},L_{E})$, where V and E are defined as before, $L_{V}$ is a function mapping a vertex v to a family set of labels,${\text{ Σ}}_{x}|\;x\in X$, such that $L_{V}:\;v\to \;{\text{Σ}}_{x}|\;\forall v\in V$, and $L_{E}$ is a function that maps an edge e to ${\text{Σ}}_{x}$ such that $L_{E}:\;v\to \;{\text{Σ}}_{x}\;|\;\forall e\in E$. Note X is the total set of labels. In our working examples of genomes $G_{1}\text{ and }G_{2}$, ${\text{Σ}}_{x}$ would correspond to unique identifiers for each chromosome in each genome (e.g. G_1_-CHRI, G_2_-CHRI, G_1_-CHRII, G_2_-CHRII). Note that an edge thus has a head and a tail. In other words, for two adjacent nodes v and w with the directed edge e, describing, v→w, the tail of an edge, termed $e_{t}$, is v and the head of an edge, termed $e_{h}$, is w.

Creating a single canonical quiver from two or more quiver representations can be formally described through morphisms. A *vertex-morphism* for a quiver is a function, $M_{V}:\;V\to Y$, that maps vertices from some vertex set V to a different vertex set Y belonging to an alternate quiver representation, $G^{\prime }=(Y,Z,L_{Y},L_{Z})$. Similarly, an *edge-morphism* is a function, $M_{E}:E\to Z$, that maps edges from some edge set E to an edge set Z belonging to the alternative quiver representation $G^{\prime }$. Therefore, the applications of $M_{V}$ and $M_{E}$ on $G_{1}\text{ and }G_{2}$ result in the transformation to a single, canonical quiver,$\;G^{\prime }$(see Fig. 1E). In this context, the canonical quiver $G^{\prime }$ is a graphical representation containing the synteny disruptions (e.g. structural variation) in $G_{1}\text{ and }G_{2}$; and the morphisms $M_{V}$ and $M_{E}$ describe either ‘unique’ genes or the merging of orthologous sequences. Acquiring $G^{\prime }$ for some set of quivers thus requires the construction of the morphisms $M_{V}$ and $M_{E}$ from the set of given quivers.

We have now described how we can obtain a single, canonical quiver $G^{\prime }$, from a set of individual quiver genome representations. $G^{\prime }$ describes disruptions of synteny within the a set of genomes which are indicative of structural variation across multiple genomes and can be obtained via the construction of vertex and edge-morphisms. In the next section, we describe our implementation of constructing these morphisms from a set of genomes through synteny-based ortholog clustering.

### 2.2 Constructing morphisms via syntenic anchors

We can construct the vertex and edge-morphisms for a canonical quiver by performing synteny-based ortholog clustering. *Ortholog clustering* aims to identify sets of corresponding orthologous sequences across a given number of genomes, and is generally obtained through some form of pairwise sequence alignment (either DNA or protein) combined with phylogenetic-inference. For constructing a canonical quiver representation, we require ortholog clusters that are syntenically supported—in other words, sequences that maximize the synteny in the surrounding region of each gene for all genomes in the cluster. We refer to these clusters as *syntenic anchors.* For example, two genes from two genomes may share high sequence similarity and thus form an ortholog cluster. However, the two genes may be located in completely different areas of the genome sharing no synteny in the surrounding regions. In the context of constructing the vertex and edge-morphisms for aligning multiple genomes, we wish to avoid forming these clusters as they will result in spurious connections of dissimilar regions across multiple genomes.

**Fig. 2.**
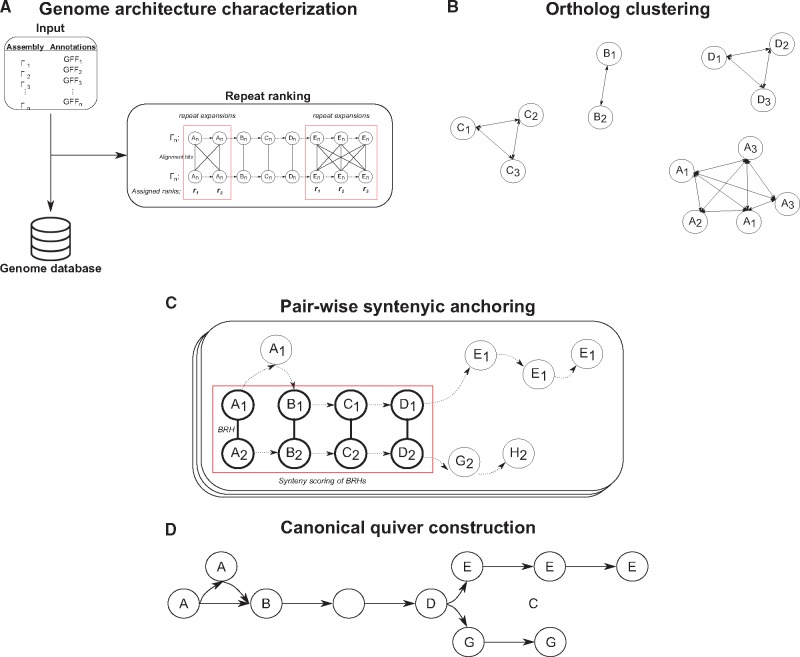
Overview of ptolemy. **(A)** Ptolemy first creates a database characterizing individual genome architectures for a given list of assemblies and their corresponding gene annotations. In this process, Ptolemy also attempts to identify repeat expansion though self-pairwise gene alignments. **(B)** BRHs are then identified via pairwise gene alignment for every pair of genomes. **(C)** Syntenic anchors are derived for each BRH by scoring the synteny of the surrounding region of corresponding genes. This is done in a pairwise fashion for every pair of genomes. **(D)** The syntenic anchors are then used to construct the canonical quiver for all genomes in the database

Figure 2 gives an overview of our procedure to identifying syntenic anchors. We present a generalized description of our approach, and exact details can be found in Supplementary Material ‘Methods’. First, we create a database describing the architecture of each genome such as chromosome content including gene sequence and location (see Fig. 2A). Genes with overlapping open reading frames are merged together into a single ‘gene unit’ whose boundaries are defined by the minimum and maximum coordinates of all overlapping open reading frames. During the database creation, we attempt to identify repeat expansions by identifying connected graphs induced from self-pairwise-gene alignments (see Fig. 2A) and assign *repeat ranks* describing the order of genes in these regions. We then identify ortholog clusters throughout all genomes in the database by identifying best reciprocal hits (BRHs) through pairwise alignments of the gene sequences for every pair of genomes (see Fig. 2B).

Syntenic anchors can then be derived from BRHs by scoring the synteny of their neighboring regions (see Fig. 2C). Similar to several synteny region finders (Drillon *et al.*, 2014; Gehrmann and Reinders, 2015; Proost *et al.*, 2012; Tang *et al.*, 2015), we use a general window scoring approach (such as nearby genes of a given position) as well as independent left and right flanking windows (nearest genes strictly upstream and downstream) which enables us to handle structural rearrangements such as translocations and inversions. We determine whether a BRH is a syntenic anchor by computing a *synteny score* for each window (see Supplementary Material ‘Methods’). Conceptually, for some defined window size, we iterate through each position upstream and downstream from a BRH and compute the difference between expected and observed synteny based on the positional displacement of neighbouring genes (see Supplementary Material ‘Methods’). In implementation, BRH’s are considered syntenic anchors if their synteny score meets a minimum threshold. A detailed description of this parameter along with how to set it can be found in the Supplementary Material ‘Methods’.

Last, for a BRH containing genes involved in a repeat expansion, we compute the syntenic score of the neighbouring regions outside of the repetitive region. The intuition is that locally repetitive regions will cause inaccurate calculations for the synteny scores for both genes that are within and around the repetitive region leading to an increase of false negative syntenic anchors. Thus, we ‘mask’ the repetitive regions and compute the synteny upstream and downstream of the region. Furthermore, we restrict the synteny scoring of repetitive genes to those that only have the same *repeat_rank* normalizing the syntenic anchors of repetitive regions to their left-most corresponding BRH.

We have described our procedure for identifying syntenic anchors using a synteny-based scoring mechanism for each BRH. The scoring mechanisms accounts for structural variation—such as translocations, inversions, and horizontal gene transfers—and consistently handles repetitive regions such as repeat expansions. With the syntenic anchors in hand, we can construct the edge and vertex-morphisms to create the canonical quiver representation for a given set of genomes. In the next section, we describe our procedure for constructing the morphisms, and hence, the canonical quiver.

### 2.3 Canonical quiver construction

We construct the edge and vertex-morphisms by merging all genes in a syntenic anchor into a single node, implicitly constructing the edge-morphism as well (see Fig. 2D). Let a syntenic anchor be represented as a family of sets, $A_{i}\;|\;i\;\in I$, where I is the total number of syntenic anchors. By merging all genes in each $A_{i}$, we construct the vertex-morphism, $M_{V}:\;v\to y\;|\;\forall v\in A_{i},\;\forall y\in \;Y$, where Y is the set of nodes in the canonical quiver, $G^{\prime }=(Y,Z,L_{Y},L_{Z})$. Concatenating the labels (e.g. chromosome identifiers) for all genes in $A_{i}$ constructs the vertex label function, $L_{Y}$. Note that the universal set of vertex labels (e.g. the union of all vertex labels in the canonical quiver) is the union of all labels in a set of genomes and the label of each vertex is therefore a subset of the universal set of vertex labels. Implicitly, we also construct the edge-morphism, $M_{V}:\;e\to z\;|\;e_{t},e_{h}\in \overset{N}{\underset{1}{\cup }}V_{n},\forall z\in \;Z$, where the tail and head of an edge, $e_{t}$ and $e_{h}$, are a vertices from one of the N genomes in the database. Conceptually, we are merging all edges whose head and tail are part of the same syntenic anchor. Similarly, the concatenation of all edge labels defined by the edge-morphism similarly leads to the construction of the edge-label function, $L_{Z}$.

In our implementation, we output the canonical quiver in a GFA-formatted file (Li, 2016). Each node is represented with the unique identifier assigned during the database or vertex-morphism construction. The *path lines* describe the original architecture of a sequence (e.g. chromosome) using the node identifiers and, hence, can be used to extract the edge and vertex labels. We additionally add a *genome line* starting with the identifier ‘G’ describing the set of sequences for each genome. The resulting GFA-formatted file is portable and can be immediately visualized in any GFA-supported graph visualizer such as Bandage (Wick *et al.*, 2015).

### 2.4 Structural variant calling using quiver representations

Structural variants are traditionally based on a reference genome, but can also be described as a family of subgraphs each describing architectural similarities and differences across a population. Recall our working example of genomes $G_{1}$ and $G_{2}$ (see Fig. 1). We can describe the structural variant as an insertion of three genes in $G_{2}$ with respect to $G_{1}$. Conversely, we can describe it as a deletion of three genes in $G_{1}$ with respect to $G_{2}$. In either case, this approach makes use of a reference-genome. However, we can also partition the canonical quiver and describe the graph as a family of subgraphs describing genomic similarities and differences as a population. For example, Genes 1–3 and 4 and 5 can form two disconnected components each describing common genomic architectures between $G_{1}$ and $G_{2}$. Genes 6–8 can also form a disconnected component but instead describe a variant in the genomic architecture between $G_{1}$ and $G_{2}$.

We identify structural variants in the canonical quiver using a two-step hybrid, reference and population-based approach. We use an inductive graph data structure (Erwig, 2001) for representing a canonical quiver enabling us to use a functional paradigm for identifying structural variants. Given a canonical quiver, $G^{\prime }=(Y,Z,L_{Y},L_{Z})$, we first define a reference architecture used to partition the quiver into a family set of subgraphs representing differences across the given collection of genomes with respect to a commonly observed population. By default, the reference architecture is obtained by computing the most common genome architecture in the canonical quiver based on the frequency of sub-populations within all edges. Specifically, for a given connected component, we obtain the label of all edges, count the number of occurrences for a given group of labels, and use the label with highest count; resulting in the most co-occurring group of genomes in the canonical quiver—similarly to obtaining the ‘most weighted path’. Optionally, the reference genome architecture can be computed using co-occurrences of sub-populations within nodes rather than edges. For more specific comparisons—e.g. comparing pathogenic to non-pathogenic genomes—users can specify a specific population as the reference architecture.

Given the label of the reference architecture, ${\text{Σ}}_{R}$, we perform a *reference-cut operation*: we remove all edges, satisfying, ${\text{Σ}}_{R}\subseteq L_{E}\left(z\right)\;|\;{\text{Σ}}_{R}\;\in \overset{X}{\underset{1}{\cup }}\Sigma_{x},\;z\in Z$, followed by the removal of all vertices satisfying, $deg^{-}\left(x\right)=deg^{+}\left(x\right)=0$. Conceptually, the reference-cut operation removes edges that are part of the reference architecture followed by nodes with no in or out-edges representing genes shared across all genomes. The result is a family of subgraphs, $\Gamma_{f}\;|\;f\;\in F$, where F is the total number of subgraphs, each representing a structural variant with respect to the reference architecture.

Each subgraph $\Gamma_{f}$, may contain additional nested structural variation that can be characterized through a recursive labeled traversal approach. As previously discussed, nested structural variation is generally missed when solely comparing against a reference genome. To characterize nested variation, we traverse through each $\Gamma_{f}$ based on *maximally labeled path traversal*: given a some starting node, $y_{1}$, and a label, ${\text{Σ}}_{x}$, we perform a depth-first search traversal to obtain the maximally labeled path, $t=(y_{1},\;y_{2},\;\ldots ,\;y_{p})$, such that $\left(y_{i},\;y_{j}\right)=\left(z_{t},\;z_{h}\right)\wedge {\text{Σ}}_{x}\subseteq L_{E}\left(z\right)|\;y_{i},\;y_{j}\in Y,\;z\in Z,\;1\leq i<j\leq p$. If we remove all edges inferred in t and subsequently remove all nodes with no in or out-edges, we recreate the reference-cut operation. Using a recursion-based implementation where a new label is used in each iteration enables us to dynamically choose a new reference architecture based on all structural similarities and differences within the population of genomes in $\Gamma_{f}$.

In our implementation, we first identify all connected components in the canonical quiver. Then, for each connected component, we compute the reference architecture, perform the reference-cut operation and tail-recursively report the maximally labeled traversals. Similarly, we store the output in a GFA file: each connected component has a corresponding GFA file describing all family of subgraphs identified and each *path line* describes a maximally labeled traversal.

### 2.5 Ptolemy implementation

All the algorithms discussed are packaged under Ptolemy and generalized in three modules. The *extraction + repeat finder* module (*E + R*) creates a database for a given set of genomes and attempts to identify repeat expansions. The *syntenic anchor* module (*SA*) performs pairwise gene alignments across all genomes in the database, obtains BRHs, and computes syntenic anchors. The *canonical quiver* module (*CQ*) constructs the canonical quiver by inferring the graph morphism functions from the computed syntenic anchors.

Ptolemy is implemented under a functional paradigm using Scala (https://www.scala-lang.org/) and released as open-source software under the GNU GPL 3 license. Binaries, source code, documentation and example datasets are available through GitHub: https://github.com/AbeelLab/ptolemy.

### 2.6 Benchmark data

We evaluated Ptolemy by aligning three different datasets representing various microbial genome architectures and populations. The *MTBC* dataset contains 24 complete assemblies from the *Mycobacterium tuberculosis complex.* The *Yeast* dataset contains 12 complete, PacBio assemblies from the *Saccharomyces sensu strictu* group—the architectures of these genomes were previously analyzed (Yue *et al.*, 2017). The *Eco + Shig* dataset contains a mixture of 20 *Escherichia coli* and *Shigella* species that are both commensal and pathogenic—the pan-genome of these organisms was previously analyzed (Lukjancenko *et al.*, 2010). The accession codes for all assemblies can be found in Supplementary Table S1. Clustering of assemblies using kmer profiles was performed with MASH (Ondov *et al.*, 2016) using kmer size of 21 and sketch size of 1 000 000. The canonical quivers were visualized using Bandage (Wick *et al.*, 2015) and internal scripts using Scala; general plots were created using ggplot2 (Wickham, 2009).

## 3 Results

Genome architectures in the microbial world can be diverse ranging from species with high sequence conservation to those with only 11% overlap in their genetic content (Lukjancenko *et al.*, 2010; Coscolla and Gagneux, 2014). We therefore evaluated the utility of Ptolemy on three microbial datasets representing the spectrum of microbial genetic diversity. The *MTBC* dataset contains complete assemblies from *M. tuberculosis* (22)*, M. canetti* (1) and *M. africanum* (1) whose genome architectures are conserved harboring little structural variation relative to other prokaryotic organisms (Coscolla and Gagneux, 2014; Ioerger *et al.*, 2009; Tsolaki *et al.*, 2004). The *Yeast* dataset contains complete assemblies of *Saccharomyces cerevisiae* (7) and *S. paradoxus* (5) which share large fraction of their synteny but are known to harbor various balanced and in-balanced complex structural variation as well eukaryotic horizontal gene transfers (Yue *et al.*, 2017). The most diverse set is the *Eco + Shig* dataset consisting of complete assemblies from *E.coli* (13), *Shigella flexneri* (3), *Shigella boydii* (2), *Shigella dysenteriae* (1) and *Shigella sonnei* (1), which have dynamic genome architectures with many complex structural variations and little overlap in their gene content (Lukjancenko *et al.*, 2010). We inspired our evaluation on previously published analyses of the structural variants and pan-genome—shared fraction of gene content across all genomes—of these datasets (Ioerger *et al.*, 2009; Lukjancenko *et al.*, 2010; Tsolaki *et al.*, 2004; Yue *et al.*, 2017).

### 3.1 Conserved genome architectures in *MTBC*

**Fig. 3.**
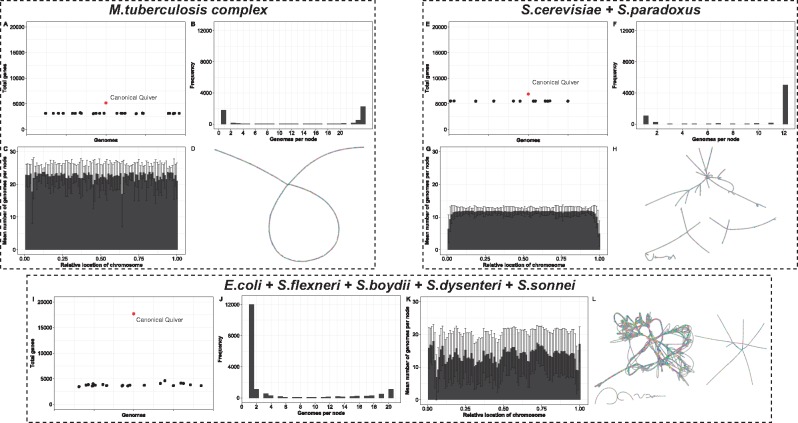
Pan-genome and canonical quiver overview of three datasets using Ptolemy. The various figures shows an overview of the pan-genome and canonical quiver derived by Ptolemy for *M.tuberculosis* genomes (top-left), *Saccharomyces* genomes (top-right) and *E.coli* and *Shigella* genomes (bottom). In general, (**A, E** and **I**) compare the total number of genomes in the canonical quiver in comparison to all genomes in the dataset. Panels (**B**, **F** and **J**) show the distribution of the number of genes shared across all genomes in the dataset. Panels (**C, G** and **K**) summarize the (B, F and J) as a function of the relative location of the chromosome. Finally, (**D, H** and **L**) show a visual representation of the canonical quivers

The graphical representation of the alignments of all genomes in the *MTBC* dataset—termed the canonical quiver—reflect previously published analyses of the pan-genomes for *Mycobacterium* species. Figure 2A–D gives overview summary of the pan-genome derived from the canonical quiver. On average, there are 1013 more genes in the canonical quiver in comparison to the gene content of the 24 assemblies (see Fig. 3A)—note that we merge overlapping reading frames into a single, maximal gene (see Section 2). Most of these genes are shared across all genomes as 76% of all genes are shared by at least half of the assemblies in the dataset (see Fig. 3B). In terms of chromosomal locations, we find that the number of genomes containing a gene is constant across the chromosome with no clear ‘hot-spots’ of unique gene content (see Fig. 3C).

**Fig. 4.**
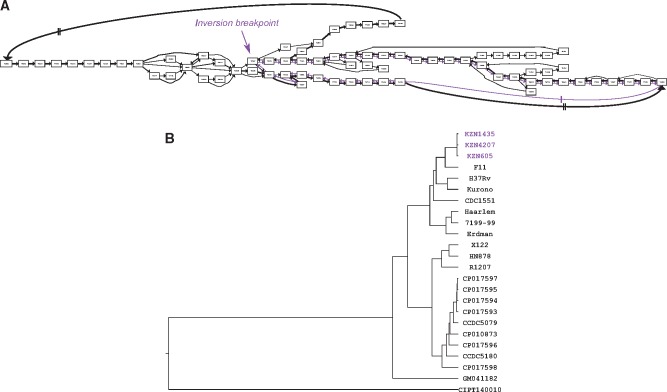
Large-scale inversion within a sub-population of *M. tuberculosis* genomes. Panel **(A)** shows a sub-graph of the canonical quiver at the breakpoint of an inversion present in three genomes. Nodes are genes and the edges describe alternative paths that different genomes take: edges are colored purple when they exclusively describe the three genomes harboring the inversion, and black otherwise. The thickness of the edge corresponds to the number of genomes traversing the paths—the more common the path the thicker the edge. **(B)** A dendogram of the hierarchical clustering of all genomes in the dataset based on Kmer profiles. The samples in purple are those harboring the large-scale inversion and which cluster together

Structural variation encoded in the canonical quivers also reflects previous analyses regarding structural variation within the *MTBC* dataset. Figure 2D visualizes the canonical quivers and is (visually) representative of how dynamic the genomes are. As shown, the canonical quiver is largely linear with a single, topological ‘loop’ in the middle. By extracting the family of subgraphs which correspond to the structural variations in the canonical quiver, we find that the loop is representative of a large-scale inversion in 3 of the 24 genomes (see Fig. 4A). Kmer-based clustering of the assemblies (see Section 2) shows that the genomes harboring the inversion also cluster together, indicative of a sub-population within this dataset (see Fig. 4B).

### 3.2 Variable genome architectures in *yeast*

The canonical quiver confirms previous reports regarding genome architectures in the *Yeast* dataset. Figure 3E–H shows an overview of the pan-gnome obtained by Ptolemy. The canonical quiver has 6919 genes, which has on average 1249 more genes in comparison to the 12 assemblies in the dataset (see Fig. 3E). Most of the genes in this dataset are shared as 80% of the gene content is present in at least half of the assemblies in the dataset (see Fig. 3F). As shown in Figure 2G, the number of genomes per gene is fairly consistent across all chromosomes except for the starting/ending sub-regions where this number sharply falls (see Fig. 3G).

**Fig. 5.**
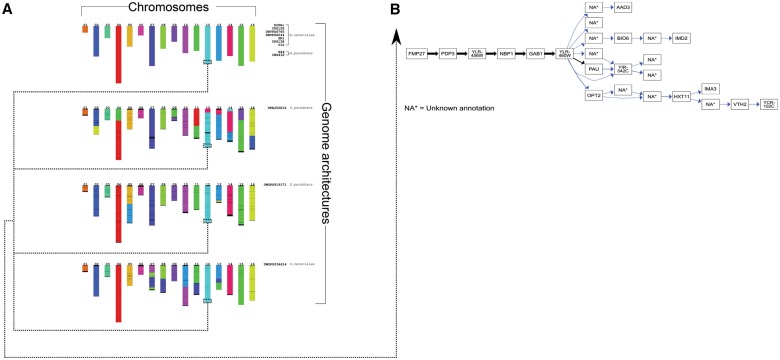
Genome-wide and sub-region-specific quiver decomposition for 12 *Saccharomyces* assemblies. Panel **(A)** shows that the decomposition of the canonical quiver results in five unique genome architectures. The first genome architecture (top-most set of chromosomes) is the most common and is largely similar to the commonly used reference genome for *S.cerevisiae*, S288C. The remaining three are much more diverse containing several translocation and inversions across the 16 chromosomes in the genome. Panel **(B)** shows a sub-region in the canonical quiver corresponding to the right sub-telomere region of chromosome XII. Black edges correspond to paths containing the reference, S288C, and blue otherwise. Note the additional structural variants present in several genomes which are absent in the reference

We were able to identify previously reported structural variation as well as additional variation likely missed due to bias in reference-based comparisons. Although linearity (e.g. synteny) is still observed throughout the quiver, Figure 3H shows various topological features reflecting several translocations and inversions. (Note the different connected components reflecting different chromosomal sequences in these organisms). By decomposing the quiver, we can reconstruct the genome architectures of the twelve genomes proposed by Yue *et al.* (2017) (see Fig. 5A). Specifically, the genome architectures for eight genomes are similar to that of the S288C, a commonly used reference genome for *S.cerevisiae* (see Fig. 5A). For the additional three genomes we find various translocations in inversions across the 16 different chromosomes (see Fig. 5A).

An example of the type of complex structural variation that exists within the *Yeast* dataset is shown in Figure 5B. The figure corresponds to a sub-graph of the canonical quiver corresponding to the alignment of the right sub-telomere region of Chromosome XII. As depicted, there are several structural variants unique to sub-populations in the dataset which are absent in the commonly used reference genome of S288C (see Fig. 5B). The bottom-most alternative path contains several genes associated to sugar and alcohol metabolism (see Fig. 5B). These genes are not only unique to 2 of 12 genomes but also contain nested structural variation which is generally missed by reference-based comparisons. An additional example is shown in Supplementary Figure S1B depicting the alignment of the right end of the sub-telomere region for chromosome VII. Yue *et al.* (2017) previously reported a tandem expansion of two paralogs, MAL31 and MAL33 (involved in the metabolism of the maltose sugar compound), for the *S.paradoxus* genome, CBS432. We find that this expansion is present—in variable length—in 9 of the 12 genomes and absent only in the *S.cerevisiae* genomes of SK1 and DBVPG6044 along with the commonly used reference, S288C (see Supplementary Fig. S1B).

### 3.3 A genomic ‘melting-pot’ in the *Eco + Shig* dataset

We observe large variations in the pan-genomes for the 20 assemblies in the *Eco + Shig* dataset. Each genome contains about 3825 genes, contrasted by the canonical quiver which has a total of 17 698 genes (see Fig. 3I). This variation is further highlighted in Figure 3J where only 18% of all genes are shared by at least half of the assemblies in the dataset. Furthermore, the number of genomes per gene is highly variable and varies throughout the chromosome (see Fig. 3K).

We investigated structural variation encoded in the canonical quiver by comparing the genome architectures of commensal and non-commensal pathogens (Lukjancenko *et al.*, 2010). The complex structure of the canonical quiver is shown in Figure 3L and highlights the dramatic variation that exists within the genomes of the *Eco + Shig* dataset. Although some linearity exists, Figure 3L shows that the canonical quiver contains many complex topological features representing various forms of structural variations, inversions, and horizontal gene transfers. (Note that a subset of these genomes contains several plasmid sequences and, hence, Figure 3L displays several connected components). In the *Eco + Shig* dataset, 9 genomes are described as commensal while the remaining 11 genomes are described as pathogenic (Lukjancenko *et al.*, 2010). We defined the reference genome architecture to the nine commensal genomes (see Section 2) and extracted the family of subgraphs representing structural variation between the two populations.

We found 50 structural variants exclusive to the pathogenic genomes of containing at least three genes and shared by at least two genomes. Among the largest structural variant is a sub-graph in the canonical quiver of about ∼ 24 genes in length that is exclusive to four *Shigella* genomes: *S.flexneri* strains 2a 301 and BS12, *S.dysenteriae* strain Sd197, and *S.sonnei* strain Ss046 (see Supplemenary Fig. S2). Closer analysis showed that this variant corresponds to the virulence-based Type III secretion system (Buchrieser *et al.*, 2000), a hallmark genetic component in pathogenic bacterial species (Coburn *et al.*, 2007).

### 3.4 Performance of Ptolemy

Although the construction of the canonical quiver can be fast—e.g. ∼10 min for 24 genomes (see Table 1)—it is important to note that the time complexity is ultimately $O(n^{2})$*.* The two most computationally intensive steps in Ptolemy are computing BRHs—which currently uses pairwise gene alignments across all pairs of genomes—and the syntenic scoring of each BRH, each which is $O(n^{2})$ (see Table 1). For the latter step, the worst case scenario is comparing highly conserved genomes (such as *M.tuberculosis* as done in this study). For this type of organisms, many genes are shared across a large fraction of all genomes and nearly every gene will have a BRH across all genomes, resulting in $n^{2}$ number of synteny scorings. Given that Ptolemy is implemented under a functional paradigm and nearly entirely immutable, these steps are easily parallelizable and currently makes use of all available CPUs. Analyzing large datasets are, in part, dependent on the number of available CPUs in a machine/cluster. As an example, we ran Ptolemy on 100 *M.tuberculosis* genomes which took a total of 1 h and 32 min using 20 CPUs.

**Table 1.** Run time of Ptolemy across three datasets

Dataset	Genomes	Module	Wall clock (min:s)	MaxMem (Gb)	CPUs
*MTBC*	24				
		E + R	0:43	0.680	1
		SA	11:35	1.35	4
		C	0:03	—	1
*Yeast*	12				
		E + R	0:45	0.694	1
		SA	5:02	1.44	4
		CQ	0:03	—	1
*Eco + Shig*	20				
		E + R	0:42	0.632	1
		SA	4:50	1.33	4
		CQ	0:03	—	1

*Note*: Ptolemy is separated in three modules: extraction + repeat finder (E + R), syntenic anchors, (SA) and construction of the canonical quiver (CQ).

## 4 Discussion

Advances in long-read sequencing technology are enabling researchers to feasibly acquire ‘complete’ assemblies for a collection of microbes. As this technology becomes more accessible, we can begin to shed light at the diversity of genome architectures across different (sub-) populations of microbial species, which has largely been hindered by limitations of reference-based computational approaches. In this article, we present Ptolemy: a reference-free method for analyzing genome architectures across a collection of microbial genomes. Ptolemy represents each genome as a labeled multi-directed graph, known as quivers. Using synteny analysis, the quivers can be merged into a single, canonical quiver representing a structural-based multiple whole genome alignment. As shown in the application of Ptolemy across three different datasets of *Mycobacterium, Saccharomyces, Escherichia* and *Shigella* species, the canonical quiver can be used to study pan-genomes as well as systematically discovering structural variants in context of (sub-)populations.

The application of Ptolemy on the three dataset shows the spectrum of genomic diversity that can exists in the microbial world. For example, the pan-genomes of the *MTBC* dataset confirm high conservation of the genome architectures of these organisms, which harbor relatively little-structural variation (Coscolla and Gagneux, 2014; Tsolaki *et al.*, 2004). Structural variants in these organisms are therefore used as lineage-specific markers (Angiuoli and Salzberg, 2011; Coll *et al.*, 2014). Specifically, we show that traversals of the canonical quiver can identify a large-scale inversion that exists within 3 of the 24 genomes (see Fig. 3); these genomes correspond to a family of highly virulent strains endemic to a sub-region in South Africa where the inversion has been previously observed (Ioerger *et al.*, 2009). It is important to note that Figure 3B shows roughly ∼2000 unique genes across the 24 assemblies. Closer analysis showed that the majority of these genes correspond to transposable insertion sequences and PE/PPE genes which are repetitive and variable across genomes (Cole *et al.*, 1998; McEvoy *et al.*, 2012; Roychowdhury *et al.*, 2015)—the latter which correspond to ∼10% of gene content in *M.tuberculosis* genomes (Cole *et al.*, 1998; McEvoy *et al.*, 2012).

For *Saccharomyces* species, sub-telomeric regions—the first/last ∼20–30 Kbp of a chromosome—are biologically relevant because they harbor gene families that heavily influence biotechnology-based phenotypes (McIlwain *et al.*, 2016; Salazar *et al.*, 2017; Yue *et al.*, 2017). However, these regions are notoriously challenging to compare across different genomes as they typically undergo gene-deletion, expansion, and reshuffling leading to highly dynamic architectures (McIlwain *et al.*, 2016; Salazar *et al.*, 2017; Yue *et al.*, 2017). Indeed, Figure 3G re-confirms previous observations of the diversity in these regions showing that the genes in the beginning/end of each chromosomes are not commonly found across all genomes. More specifically, Figure 4B shows the alignment of the right sub-telomeric region of chromosome XII across all genomes highlighting nested-structural variation unique to sub-populations in the dataset.

Expectedly, the results obtained in the *Eco + Shig* dataset dramatically differs to those of the *MTBC* and *Yeast* dataset. We observe a significant lower number of genes shared across all genomes similar to those previously reported (see Fig. 2I and J) and find more complex structural variation in the canonical quivers (see Fig. 2D, H and L). Such dynamic genome architectures can complicate comparative studies (Darling *et al.*, 2010; Lukjancenko *et al.*, 2010). Our ability to identify structural variation—specifically between commensal and pathogenic strains—highlights the viability of Ptolemy in different microbial populations.

The accuracy of the Ptolemy is depended on the accuracy of the gene annotations in a given dataset. Ptolemy only compares the gene sequences and is therefore sensitive to annotations errors. More specifically, annotation errors can lead to false negative merging of nodes inducing false positive structural variants. This is shown in Supplementary Figure S1A where the upper-most path of the alignment in the right sub-telomeric region of chromosome V is likely caused by gene annotations errors: the sum of the size of the two adjacent TOG1 annotations is approximately the same as the size of the TOG1 annotation in the bottom, adjacent path. Therefore, the alternative path will be identified as a structural variant although it is likely that this is the same sequence present in the remaining genomes in the dataset. We acknowledge that annotating genomes is an error-prone process and often requires manual curations (Klimke *et al.*, 2011; Poptsova and Gogarten, 2010). For this reason, care should be taken when comparing genomes of unknown annotation quality and we recommend ensuring genomes that are jointly analyzed are annotated through the same process. Nevertheless, we were still able to construct pan-genomes and identify structural variants that agree with previous published studies despite using genomes sequenced and annotated by different groups and pipelines (see Figs 3–5).

Future work could use a two-step alignment process: syntenic-anchoring followed by local-realignments of nodes. This is primarily to refine alignments of repetitive sequences, especially those involved/nearby repeat expansions. As discussed in the Section 3*,*Supplementary Figure S1B shows a sub-graph of the canonical quiver of *Yeast* dataset representing the alignment of right sub-telomeric region of Chromosome VII. We show that there is a tandem expansion of variable length for two paralogous genes across 9 of the 12 genomes. In this alignment, the right-flanking genes, PAU, COS2 and COS6, are present in other sub-populations and are considered BRHs but Ptolemy considers them unique for most of the genomes. This is largely due to difficulties in scoring the synteny in the surrounding region heavily influenced by the downstream repeat expansion as well as the different genes present upstream in each genome. Therefore, a two-step approach may first build the canonical quiver and followed by a traversal seeking to re-score the synteny of genes that are considered unique but possess BRHs in some defined neighborhood of a local subgraph.

## 4 Conclusion

Advancing sequencing technology is expanding our knowledge of the genetic diversity in microbial populations. Lacking are computational methods that can simultaneously compare multiple assemblies without restricting analysis to only ‘similar’ genomes. Here, we show that Ptolemy is a flexible method that can systematically identify structural variation across a collection of assemblies while providing insights in the population structure and pan-genome of the collection—all without the need of a reference. Ptolemy tackles long-standing challenges in comparative genomics including independence from a reference genome, characterization of complex structural variation as sub-populations, and viability in studying both conserved and highly dynamic genomes. The work presented here is a step forward for studying the genetic diversity that is yet to be characterized in the microbial world.

## Supplementary Material

Supplementary Data
