# Supplementary material for: Approximate, simultaneous comparison of microbial genome architectures via syntenic anchoring of quiver representations
Source: Bioinformatics. 2018 Sep 8;34(17):i732–42. doi: 10.1093/bioinformatics/bty614 (PMC6129293; doi:10.1093/bioinformatics/bty614)
Supplement: Supplementary Data [file bty614_supplementary_materials.docx]

Supplemental Materials

Contents

[1 Supplemental methods 2](#_Toc517012279)

[2.1 Constructing morphisms via syntenic anchors 2](#_Toc517012280)

[2.2 Parameter tuning 6](#_Toc517012281)

[2.3 References 8](#_Toc517012282)

[2 Supplemental Tables and Figures 9](#_Toc517012283)

[2.1 Supplemental table 1. 9](#_Toc517012284)

[2.2 Figure S1. 10](#_Toc517012285)

[2.3 Figure S2. 11](#_Toc517012286)

# Supplemental methods

## Constructing morphisms via syntenic anchors

Our first step of identifying syntenic anchors is to create a database characterizing all genes in each genome with contextual information as well whether a gene belongs to a potential repetitive region. For every genome, we iterate through the corresponding GFF-formatted file in ascending location and assign a unique global positive integer value for every entry whose feature type is labelled as “gene” and store the unique identifiers in a sorted array. Genes with overlapping open reading frames are merged together into a single, “gene unit” whose boundaries are defined the minimum and maximum coordinates of all overlapping open reading frames involved. The rank of a gene, $rank(x)$, corresponds to the “position” of a gene in a sequence (e.g. first gene will have $rank\left( x \right)=1)$. Since we are comparing multiple genomes, this information is globally stored in four hash tables: hash table Y storing information regarding the different chromosomes per genome, hash table Y' storing which genome each chromosome belongs, hash table Z storing all genes in ascending order per chromosome, and hash table Z' storing which chromosome a gene belongs to. In implementation, we iterate through each sequence and subsequently process each gene enabling us to create *Y* and *H* in a single iteration through the assembly. During the iteration, we also extract the corresponding DNA sequence of each gene based on the coordinates provided in the GFF-file and store the information in a FASTA-formatted file whose sequence identifier is the assigned unique integer value.

After processing each genome, we attempt to identify repetitive regions—such as repeat expansions—by identifying connected graphs induced from self-pairwise-gene alignments. Genomes generally contain sets of genes that are repetitive sharing high sequence similarity to other sequences other than itself. Whole genome aligners often face challenges when characterizing sequences undergoing copy number variation (e.g. paralogs and repeat expansions). To properly construct the vertex and edge-morphisms of the canonical quiver that handle such regions, we take a graph and rank-based approach: assume a genome made entirely unique genes, i.e. no gene shares high sequence similarity other than to itself, of size *n*. If we align the set of gene sequences to itself and create an undirected edge for every alignment above some quality threshold, we would obtain an undirected graph, $G=\left( V,E \right),$ where the vertex set is the set of all genes, for which hold that, $\left| V \right|=2n$, and $v,w \in E | i.f.f. v=w$. If we remove all edges where $v,w \in E \wedge v=w$ and all vertices that satisfy ${deg}^{-}\left( x \right)={deg}^{+}\left( x \right)=0$—where ${deg}^{-}(x)$ and ${deg}^{+}(x)$ are the out and in-degree of a vertex, respectively—we obtain an empty graph such that $V=E=\emptyset$. Applying this procedure to a genome with repetitive sequences will result in an undirected graph with disconnected components and can therefore be used as proxies for identifying repetitive regions.

In implementation, we use *minimap2* (Li, 2017) with the *‑‑X* parameter to remove self-alignments to perform pairwise gene alignments using the database constructed for a set of genomes. We additionally remove alignments satisfying $dist\left( x,y \right)>r$ where $dist\left( x,y \right)$ is set to $\left| rank\left( x \right)-rank\left( y \right) \right|$ if both genes are from the same sequence and $\infty$ if they are not; *r* is the maximum distance allowed between any two alignments (default is set to 10, more details for all parameters can be found at the end of this section). Furthermore, we characterize each gene in the repeat-induced graph with a *repeat_rank* corresponding to the index of a gene after sorting all genes in the graph with their original genome rank—note that a gene that is not involved in repetitive region has a $repeat\_rank$ set to 1. Thus, for every genome, we identify sets of genes that are locally repetitive along with the order of which they appear in the region.

We identify syntenic anchors by scoring the synteny of best reciprocal hits. Syntenic anchors are, by definition, syntenic regions, and many computational methods have been developed to identify them. Our approach of finding syntenic anchors is inspired from synteny-finding methods with variations to accommodate our general purpose of obtaining the edge and vertex-morphisms of a canonical quiver. More specifically, we start by identifying *best reciprocal hits* (BRHs): two ORFs,$o$ and $o^{'}$, are BRHs if a high-quality alignment exists between $o$ and $o'$ when aligning a query genome $G$ to a target genome $G'$ and *vice versa*. BRHs are widely used to identify orthologous genes between genomes. Similarly, we use *minimap2* to perform pairwise gene alignments using a kmer size (*--k* parameter) of 11 and remove alignments satisfying $\frac{min\_size \left( o,o^{'} \right)}{max\_size (o, o^{'})}>\gamma$, where $min\_size$ and $max\_size$ extract the minimum and maxium size of two sequences, respectively, and $\gamma$ is the minimum sequence ratio set to 0.75 by default.

We then derive syntenic anchors by scoring BRHs based on the shared synteny of neighbouring genes—similarly used in several synteny region finders (Proost *et al.*, 2012; Drillon *et al.*, 2014; Tang *et al.*, 2015; Gehrmann and Reinders, 2015). We utilize a combination of a general window (such as nearby genes of a given position) and independent left and right flanking windows (nearest genes strictly downstream and upstream of a given position). This enables us to handle structural rearrangements such as translocations and inversions. The windows are used to compute the *syntenic vectors* which contains information about the differences of the positional displacement of nearby ORFs under a perfect, one-to-one synteny scenario in comparison to the observed displacement. Conceptually, for some defined window size, we iterate through each position upstream and downstream from a BRH and calculate the maximum displacement based on the locations of their corresponding BRHs. Formally, for a BRH containing ORFs $o_{i}$ and $o_{j},$the positional displacement of an ORF *x* and *y* positions away in respects to $o_{i}$ and $o_{j}$ is defined as:

$$\Psi\left( o_{i},o_{j},x,y \right)=max\left\{ \begin{aligned} \left| dist\left( o_{\left( i+x \right)},o_{i} \right)-\varphi\left( o_{\left( i+x \right)},o_{j} \right) \right|, \\ \left| {dist(o}_{\left( j+y \right)},o_{j})-\varphi\left( o_{\left( j+y \right)},o_{i} \right) \right| \end{aligned} \right\}$$

In short, the left-side of the $max$ argument computes the theoretical displacement under a one-to-one synteny scenario and the right-side computes the observed displacement. In particular, the function, $\varphi\left( o_{a},o_{b} \right)$, controls instances when multiple or no local BRHs exist within the flaking window of size *f* for ORF $o_{a}$ in reference to ORF $o_{b}$ (*f* is set to 10 by default) and is defined as:

$$\varphi\left( o_{a},o_{b} \right)= \left\{ \begin{aligned} \min_{l\in L} \{dist\left( l, o_{b} \right)\}, &|L|\geq0 \\ -\alpha, \left| L \right|=0 \end{aligned} \right.$$

*L* is a set containing all BRH’s of gene $o_{a}$ originating from the same sequence as gene $o_{b}$ and is within *k* positions away such that $\left| rank(x)-k \right|\leq f$. If *L* is empty, then $\varphi\left( o_{a},o_{b} \right)$is set to $\alpha$ the *BRH penalty* (set to 5 by default). The syntenic vectors for a BRH can thus be created follows:

$$V_{L}=[\Psi\left( o_{i},o_{j},x-f,y-f \right), \ldots\Psi\left( o_{i},o_{j},x-2,y-2 \right), \Psi\left( o_{i},o_{j},x-1,y-1 \right)]$$

$V_{R}=[\Psi\left( o_{i},o_{j},x+1,y+1 \right),\Psi\left( o_{i},o_{j},x+2,y+2 \right), \ldots\Psi\left( o_{i},o_{j},x+f,y+f \right)$]

$V_{L}'=[\Psi\left( o_{i},o_{j},x-f,y+f \right), \ldots\Psi\left( o_{i},o_{j},x-2,y+2 \right), \Psi\left( o_{i},o_{j},x-1,y+1 \right)$]

$V_{R}'=[\Psi\left( o_{i},o_{j},x+1,y-1 \right),\Psi\left( o_{i},o_{j},x+2,y-2 \right), \ldots\Psi\left( o_{i},o_{j},x+f,y-f \right)$]

Note that we create $V_{L}$ and $V_{R}$ separately to properly compute the synteny at the breakpoints of chromosomal translocations and horizontal gene transfers. Similarly, $V_{L}'$ and $V_{R}'$ are the syntenic vectors under a scenario of an inversion structural rearrangement.

The *synteny score* summarizes the vectors as a single score defined as the minimum of the mean displacement of each syntenic vector. Note that these vectors are only defined for genes that are at least *f* positions away from the boundaries of a sequence. In situations where this is not true, we obtain the maximum reachable positions upstream and downstream of a BRH, termed *max_left* and *max_right*, where $max\_left$ and $max\_right$ are both $\leq\frac{f}{2}$, and construct a general syntenic vector defined as:

$$V_{G}=[\Psi\left( o_{i},o_{j},x-max\_left,y-max\_left \right), \ldots, \Psi\left( o_{i},o_{j},x- max\_right,y-max\_right \right)]$$

In this situation, the syntenic score is set to the mean displacement in $V_{G}$.

Lastly, when scoring the syntenic score for a BRH containing genes involved in a repetitive region as previously discussed, the syntenic vectors are computed starting from the left and right-most gene in the repetitive region. The intuition is that locally repetitive regions will cause inaccurate calculations for the synteny scores for both genes that are within and around the repetitive region leading to an increase of false negative syntenic anchors. Thus, we “mask” the repetitive regions and compute the synteny upstream and downstream of the region. Furthermore, we restrict the synteny scoring of repetitive genes to those that only have the same *repeat_rank* normalizing the syntenic anchors of repetitive regions to their left-most corresponding BRH.

In implementation, we group the BRH’s of every gene based on the originating genome identifier and for each genome we retain the corresponding gene with the smallest synteny score with the restriction that $synteny score\leq S$, where $S$ is the maximum displacement threshold as a function of the window size set to $\beta\cdot f$ ($\beta$ is set to 0.5 by default). Every gene can therefore have at most one BRH for each genome. The superset of these pairs thus forms the syntenic anchors which are used to create the edge and vertex-morphisms for the canonical quiver.

## Parameter tuning

All parameters introduced in this section influence the scoring of BRHs. The two most important parameters are the size of the flanking window, *f*, and the maximum tolerable difference between the expected and observed frequency, $\beta$. Changing the *f* parameter influences the size of the surrounding region when computing the synteny score for a BRH. The default value of 10 was based on observed synteny of *Mycobacterium* and *Saccharomyces* genomes, which are highly syntenic (Tsolaki *et al.*, 2004; Yue *et al.*, 2017). Note that *f* likely needs to be decreased when analysing less syntenic genomes, such as those that harbour little overlap in their gene content. The maximum tolerable difference of the expected and observed synteny for a given BRH is controlled by parameter $\beta$, which is a function of the flanking window size. Increasing $\beta$ allows a higher discrepancy between the expected and observed synteny of a BRH in a flanking window of size *f*, while decreasing $\beta$ enforces the surrounding region for a BRH to be highly syntenic. The default value of 0.5 for $\beta$ was based on the sub-telomeric regions of *Saccharomyces* genomes where the synteny sharply decreases towards the ends of the chromosomes due to a high density of structural variation (Tsolaki *et al.*, 2004; Yue *et al.*, 2017; Salazar *et al.*, 2017).

The remaining parameters controls the quality of the alignments. When identifying repetitive regions, the parameter, *r*, determines the maximum distance allowed before partitioning the repeat-induced graph into two separate regions. This influences proper alignments of genes involved in repetitive regions such as repeat expansions. The default value of 10 was based on a characterized tandem duplication of two adjacent paralogous sequences (Yue *et al.*, 2017). Repetitive sequence can induce spurious hits during pairwise sequence alignments. One common metric used to remove such instances is to require a minimum sequence coverage threshold for all gene alignments (Ward and Moreno-Hagelsieb, 2014). However, gene annotations can be noisy and different genomes may report different lengths despite possessing the same sequence (Poptsova and Gogarten, 2010; Klimke *et al.*, 2011). Therefore, the $\gamma$ parameter controls the maximum size discrepancy in a gene alignment as a ratio of the minimum over the maximum gene size for any two sequences. The default value of 0.75 was based on observed spurious alignments of repetitive sequences and discrepancies in the annotation lengths in *Saccharomyces* assemblies (Yue *et al.*, 2017).

## References

Drillon,G. *et al.* (2014) SynChro: A fast and easy tool to reconstruct and visualize synteny blocks along eukaryotic chromosomes. *PLoS One*, **9**.

Gehrmann,T. and Reinders,M.J.T. (2015) Proteny: Discovering and visualizing statistically significant syntenic clusters at the proteome level. *Bioinformatics*, **31**, 3437–3444.

Klimke,W. *et al.* (2011) Solving the problem: Genome annotation standards before the data deluge. *Stand. Genomic Sci.*, **5**, 168–193.

Li,H. (2017) Minimap2: fast pairwise alignment for long nucleotide sequences. *arXiv*, 2–5.

Poptsova,M.S. and Gogarten,J.P. (2010) Using comparative genome analysis to identify problems in annotated microbial genomes. *Microbiology*, **156**, 1909–1917.

Proost,S. *et al.* (2012) i-ADHoRe 3.0-fast and sensitive detection of genomic homology in extremely large data sets. *Nucleic Acids Res.*, **40**.

Salazar,A.N. *et al.* (2017) Nanopore sequencing enables near-complete de novo assembly of Saccharomyces cerevisiae reference strain CEN.PK113-7D. *FEMS Yeast Res.*, **17**.

Tang,H. *et al.* (2015) SynFind: Compiling syntenic regions across any set of genomes on demand. *Genome Biol. Evol.*, **7**, 3286–3298.

Tsolaki,A.G. *et al.* (2004) Functional and evolutionary genomics of Mycobacterium tuberculosis: insights from genomic deletions in 100 strains. *Proc. Natl. Acad. Sci. U. S. A.*, **101**, 4865–4870.

Ward,N. and Moreno-Hagelsieb,G. (2014) Quickly finding orthologs as reciprocal best hits with BLAT, LAST, and UBLAST: How much do we miss? *PLoS One*, **9**.

Yue,J.X. *et al.* (2017) Contrasting evolutionary genome dynamics between domesticated and wild yeasts. *Nat. Genet.*, **49**, 913–924.

# Supplemental Tables and Figures

## **Supplemental table 1**.

Genbank and refseq accession for all genome assemblies used in this study. For the Yeast dataset, all assemblies where downloaded from <https://yjx1217.github.io/Yeast_PacBio_2016/data/> downloaded on August 3, 2017.

| **Dataset** | **Genbank/Refseq accession** |
| --- | --- |
| *Eco+Shig* |  |
|  | GCF_000005845.2 |
|  | GCF_000007445.1 |
|  | GCF_000017765.1 |
|  | GCF_000013305.1 |
|  | GCF_000010245.2 |
|  | GCF_000017985.1 |
|  | GCF_000019425.1 |
|  | GCF_000022665.1 |
|  | GCF_000021125.1 |
|  | GCF_000026245.1 |
|  | GCF_000026265.1 |
|  | GCF_000026305.1 |
|  | GCF_000022345.1 |
|  | GCF_000006925.2 |
|  | GCA_000007405.1 |
|  | GCA_000013585.1 |
|  | GCA_000020185.1 |
|  | GCA_000012025.1 |
|  | GCF_000012005.1 |
|  | GCA_000092525.1 |
| *MTBC* |  |
|  | GCF_000195955.2 |
|  | GCF_000008585.1 |
|  | GCF_000016925.1 |
|  | GCF_000023625.1 |
|  | GCF_000153685.2 |
|  | GCF_000154585.2 |
|  | GCF_000184005.1 |
|  | GCF_000184025.1 |
|  | GCF_000184045.1 |
|  | GCF_000270365.1 |
|  | GCF_000400615.1 |
|  | GCF_000331445.1 |
|  | GCF_000350205.1 |
|  | GCF_000954155.1 |
|  | GCF_002116755.1 |
|  | GCF_002116775.1 |
|  | GCF_002116795.1 |
|  | GCF_002116835.1 |
|  | GCF_002116855.1 |
|  | GCF_002116815.1 |
|  | GCF_000828995.1 |
|  | GCF_000154605.2 |
|  | GCF_000253355.1 |
|  | GCF_000253375.1 |


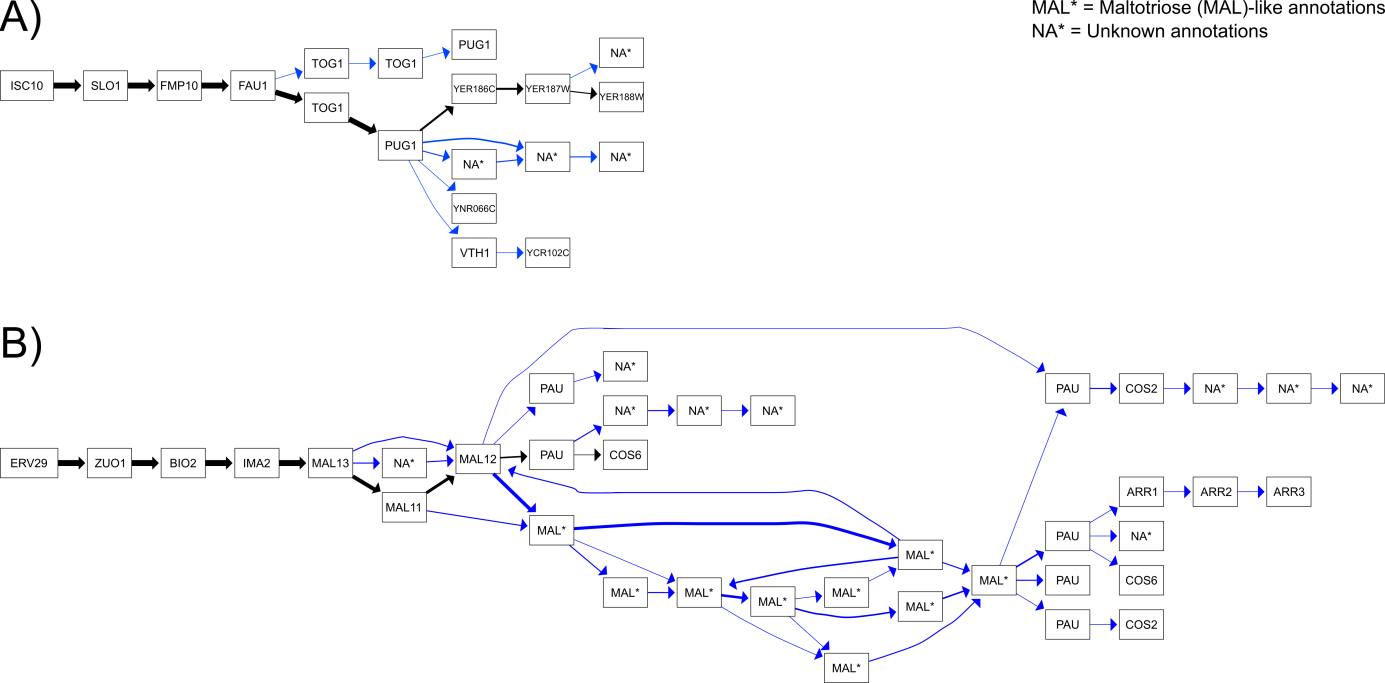


## **Figure S1.**

Sub-telomere alignment containing annotation and repeat-induced errors. (A) shows a sub-graph in the canonical quiver representing the alignment of the right-sub-telomeric region in chromosome V for 12 Saccharomyces assemblies. Similarly, (B) shows the alignment of the right sub-telomeric region for chromosome VII which contains a repeat expansion of MAL-like genes. For both graphs, black edges denotes paths containing the reference genome, S288C, and blue otherwise. The weight of the edge corresponds to number of traversing through a given edge.

##
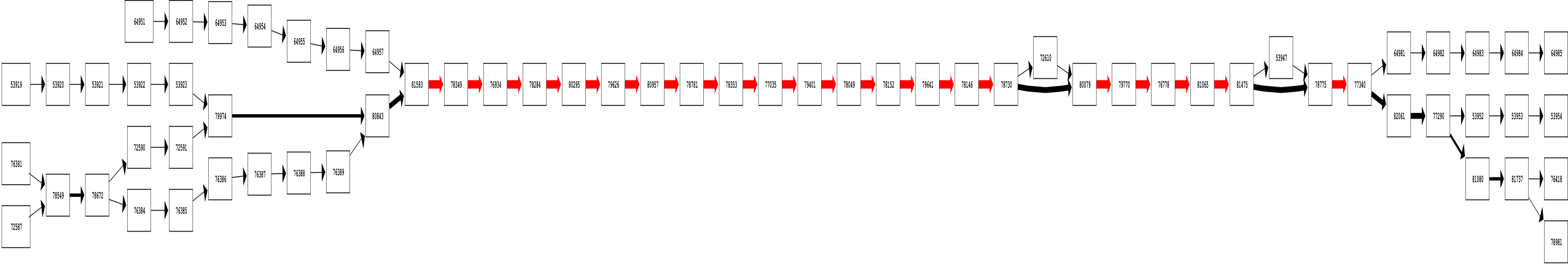
**Figure S2.**

Sub-telomere alignment containing annotation and repeat-induced errors. (A) shows a sub-graph in the canonical quiver representing the alignment of the right-sub-telomeric region in chromosome V for 12 *Saccharomyces* assemblies. Similarly, (B) shows the alignment of the right sub-telomeric region for chromosome VII which contains a repeat expansion of MAL-like genes. For both graphs, black edges denotes paths containing the reference genome, S288C, and blue otherwise. The weight of the edge corresponds to number of traversing through a given edge.
